# Towards the Development of Functionalized Polypyridine Ligands for Ru(II) Complexes as Photosensitizers in Dye-Sensitized Solar Cells (DSSCs)

**DOI:** 10.3390/molecules190812421

**Published:** 2014-08-15

**Authors:** Adewale O. Adeloye, Peter A. Ajibade

**Affiliations:** Department of Chemistry, University of Fort Hare, P.M.B. X1314, Alice 5700, South Africa; E-Mail: pajibade@ufh.ac.za

**Keywords:** synthesis, ruthenium(II) polypyridines, polyaromatic, intermolecular charge transfer, photophysical properties, electro-redox properties, DSSCs

## Abstract

A number of novel ruthenium(II) polypyridine complexes have been designed and synthesized for use as photosensitizers in dye-sensitized solar cells (DSSCs) due to their rich photophysical properties such as intense absorption, long-lived lifetimes, high emission quantum yields and unique redox characteristics. Many of these complexes exhibit photophysical behavior that can be readily controlled through a careful choice of ligands and/or substituents. With this perspective, we review the design and general synthetic methods of some polypyridine ligands based on bipyridine, phenanthroline, terpyridine and quaterpyridine with/without anchoring groups with a view to correlate functionality of ligand structures with the observed photophysical, electroredox and power conversion efficiency of some examples of Ru(II) polypyridyl complexes that have been reported and particularly used in the DSSCs applications. The main interest, however, is focused on showing the development of new polypyridine ligand materials containing long-range electron transfer motifs such as the alkenyl, alkynyl and polyaromatic donor functionalities.

## 1. Introduction

The recent interest generated by the study of Ru(II) polypyridine complexes has stimulated the growth of several branches of pure and applied chemistry. These complexes are still to be reckoned with for the key roles they play in the development of photochemistry, photophysics, photocatalysis, electrochemistry, photo-electrochemistry, electro-chemiluminescence, electron and energy transfer processes. In the area of photochemistry, the forecasts for the next 50 years predict that human energy needs are likely to double while fossil energy reserves are shrinking. In an attempt to reduce the use of fossil fuels which are limited and contribute heavily to global warming, scientists are looking for other sustainable energy sources to serve our future energy needs. Of these, solar energy looks promising because the Earth receives more energy from the Sun in one hour than is used by all humanity in the course of an entire year [1].

Much research has gone into producing efficient solar cells, the devices which convert sunlight into electricity through a process called the photovoltaic effect. Solar energy is an environmentally friendly, abundant and also free renewable energy source. Therefore, direct photo-conversion of solar energy by using photovoltaic technology is increasingly recognized as a viable high-tech solution to the growing energy challenge [2].

Currently, solar cells are made largely from inorganic semiconductors such as highly purified silicon, similar to those used in computer chips, but these inorganic semiconductors are expensive because they require high purity crystals and need to be fabricated under vacuum. Therefore, researchers are seeking new inexpensive alternatives. Research into organic and organometallic molecules (in the form of both small-molecules and macromolecules), has recently shown great promise. In recent years, many attempts have been made to develop new sensitizers for practical usage. Sensitizers based on ruthenium complexes [3,4,5,6,7,8,9,10,11,12,13], metal free organic dyes [14,15,16,17] and porphyrins [18,19,20,21,22] were developed and used as efficient sensitizers for DSSCs. Among them, ruthenium and porphyrin based dyes have reached power conversion efficiencies of more than 10%. On one hand, ruthenium complex dyes (e.g., **N3**, **N719** and **BlackDye**) were argued to be unsuitable due to cost effectiveness and environmental concerns, as ruthenium is a rare and expensive metal which limits the potential for wide applications of these complexes. Thus, porphyrin dyes have been put forward as an alternative to ruthenium complexes due to advantages such as the diversity of their molecular structures, high molar extinction coefficient, simple synthetic route, low cost, and environmental friendliness [23].

**Figure 1:**
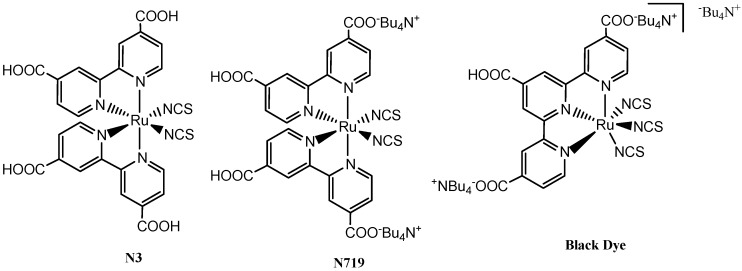
Structures for benchmark sensitizers **N3**, **N719**, and **BlackDye** for DSSC applications.

Among ruthenium(II) polypyridyl complexes, **N719** and **BlackDye** are grouped among the “champion dyes” (*i.e.*, dyes that generate a PCE > 10% in the DSSC, Figure 1). The photovoltaic efficiencies of this group of dyes, however, are still not large enough to meet the requirements for commercial use. The main disadvantage of **N719** for example, is the absence of strong absorption towards longer wavelengths in the visible spectrum. Although **BlackDye** has an absorption extending into the near-IR region with a photo-response up to 920 nm, the efficiency of its associated solar cell is not as high as what is desirable. Specifically, metal complex sensitizers used for DSSCs have two major ligands; the anchoring ligands are required for the complex adsorption on the semiconductor surface and the ancillary ligands, which are responsible for the tuning of the overall properties of the complex. Based on these, the photovoltaic performances in terms of conversion yield and stability depended on tuning of the ancillary ligands. Nguyen, *et al*. [24] showed that the current set of commercially relevant dyes are susceptible to degradation over time due, at least in part, to the loss of the monodentate, labile NCS^−^ ligands. Thus, in a complicated DSSC system, other factors which include the molecular structure of complexes, number and position of anchoring ligands, morphology of semiconductor, electron injection, sensitizer regeneration processes, electrolyte regeneration processes, charge recombination as well as back transfer processes may significantly contribute to lowering the good performance of the DSSC [25,26].

**Figure 2:**
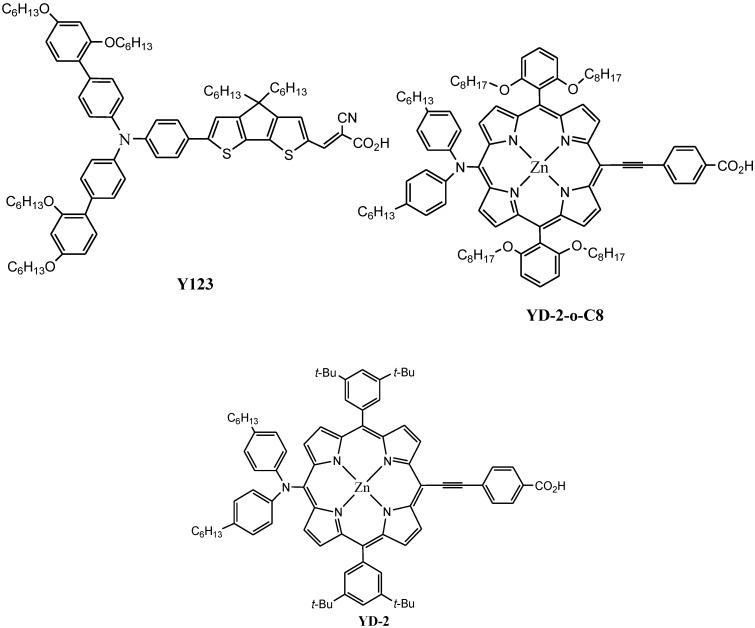
Co-sensitization of **Y123** and **YD-2-o-C8** (world record holder with a PCE > 12%).

Recently, Gratzel, *et al*. [22] reported a d-π-A based zinc porphyrin dye (**YD2-o-C8**) and achieved solar to electric conversion efficiency of up to 12.3% under standard global AM 1.5 solar conditions with enduring stability. **YD2-o-C8** is the best porphyrin dye so far reported, other than the ruthenium based dyes. However, the molar extinction coefficients of **YD2-o-C8** (Figure 2), and its derivatives in the region of 500–600 nm as well as in the long wavelength region are still considered low and hence the decrease in the light harvesting abilities. Many groups are trying to overcome this problem by introducing various substituents but there has been very little change in the molar absorption to date [27,28,29,30,31].

Recent works have shown that in order to obtain high PCE values in DSSC, the dyes must conform to essential design requirements to function efficiently [32,33,34]. These requirements include: a complete charge-separation, absorption of solar radiation wavelengths in the visible or near-IR region, appropriate ground and excited state redox potentials, presence of an anchoring group such as the carboxylic or phosphonic acid needed to bind strongly to the TiO_2_ semiconductor, and efficient electron injection into the TiO_2_ conducting band. This must be accompanied by a rapid electron transfer from the dye to the nanocrystalline semiconductor (TiO_2_) compared to the dye ground state decay [35]. According to Robson, *et al*. [36], in order to facilitate effective electron injection at the TiO_2_ surface as shown in **N3** dye (HOMO and LUMO energy levels), the distance of the donor chromophore group from the acceptor ligand should to be held in close proximity, leading to light-induced charge-separation. The results from this set-up are expected to lead to electronic asymmetry lowering of the degeneracy of the frontier orbital energy levels, which subsequently should increase the number of non-degenerate allowed transitions and hence result in a better light absorption.

### 1.1. Basic Operating Principles of Dye Sensitized Solar Cells (DSSCs)

A typical DSSC consists of two glass plates coated with a transparent conductive oxide layer. The working electrode is covered with a film of a dye-sensitized substance (typically Ru(II) polypyridyl complexes), and the counter electrode is coated with a catalyst. Both plates are sandwiched together with the gap between them filled by an electrolyte. Light absorption is carried out by dye molecules in which the absorbed photons cause dye photo excitation to release an electron rapidly to the semiconductor. The injected electrons then hop through the colloidal TiO_2_ particles to reach the collector. Following this, the electron passes through an outer circuit to reach the other transparent conductive oxide layer at the counter electrode, ultimately doing electrical work for the user. Finally, the electron is transferred to the electrolyte where it reduces the oxidant and the reduced form reduces the excited dye to the ground state and completes the circuit [37].

### 1.2. Approaches to Development of Functionalized Polypyridine Ligands for Ruthenium Complexes

The development of DSSC devices has been greatly influenced over a considerable amount of time by the chemical architecture of both the condensed phase and molecular components from which it is made. Synthetically, it has been established that varying the conjugation level of the polypyridine ligand systems through introduction of suitable groups as substituents in the periphery of the ligand tend to induce and tune both spectra and redox properties of the corresponding molecules. Efficient sensitizers have been found among coordination compounds of the general structure ML2X2, where L stands for functionalized bipyridyl or phenanthrolyl, M = Ru(II) and X represents a halide, cyanide, thiocyanate, acetyl acetonate or thiocarbamate [38].

From the first reports of the photochemical processes of the DSSC, surface/molecular interactions and the role of hetero supramolecular assembly have become apparent as the underlying principles of this and related PEC devices. It is therefore imperative to further examine and understand the chemical properties and synthetic approaches used to obtain materials for the construction of dyes for DSSCs. It has been reported that the choice of ligand, structure and/or substituents on nitrogen-based coordinated ligands are paramount in order to influence the photophysical and electro-redox properties of the coordinated ruthenium complexes, which may lead to enhanced incident photon to current efficiency (IPCE) in the resulting dye sensitized solar cells.

The most studied complexes are based on [Ru(bpy)_3_]^2+^ (bpy = 2,2'-bipyridine), as they have been shown to have long-lived excited states at room temperature that originate from their metal-to-ligand charge-transfer (MLCT) transitions [39,40,41,42,43,44,45,46,47]. According to Keene and co-workers, bpy-based complexes induce chirality at the metal centre, and separation of the enantiomers in polymetallic complexescan be complicated, leading to the development of achiral tridentate 2,2':6',2"-terpyridine (tpy)-type ligands [48,49].

## 2. Syntheses and Trends in Dye Sensitizer Development

In the last two decades, the development of syntheses of different arrays of dyes, particularly the ruthenium(II) polypyridyl complexes has intensified and employed in the conversion of solar energy to electricity. The pioneer dye referred to as **N3** dye (4,4'-dicarboxyl-2,2'-bipyridyl-ruthenium(II)-dithiocyanate) as well as some of its derivatives have shown great promise with a highest solar-to-energy conversion efficiency of about 11% compared to silicon-based photovoltaic cells with 16% conversion efficiency. The major drawback of **N3** dye is in its lack of absorption in the red region of the visible spectrum. The molecular designs of ruthenium polypyridyl complexes as photosensitizers for nanocrystalline TiO_2_ solar cells that can absorb visible light of all colours presents a challenging task. The most suitable dye is expected to have suitable ground- and excited- state redox properties so that the two key electron-transfer steps (charge injection and regeneration of the dye) occur efficiently. It has thus been difficult to fulfill both requirements simultaneously during the design of a charge transfer (CT) sensitizer.

Numerous attempts have been made to molecularly engineer ruthenium sensitizers to broaden the absorption band and increase the molar extinction coefficient. One successful approach taken by various authors has been the functionalization of one arm of the 4,4'-dicarboxylic 2,2'-bipyridyl anchoring ligands in the **N3** ruthenium sensitizer with different arrays of highly conjugated ancillary ligands, for example, 2-hexylthiophene (**HRS-1**) [3,50], dialkylaminobenzene [51], alkyl bithiophene [52], alkoxybenzene, 2-(4-*tert*-butyloxyphenyl)ethylene [53,54], dipyridylamine [55], 3,4-ethylenedioxy- thien-2-yl)vinylene [56], 3,5-di-*tert*-butylbenzene [57], and 4,4-bis(4-*tert*-butylstyryl) [58], to form new ruthenium(II) polypyridyl dyes in order to enhance both the molar extinction coefficient and red-shift of the metal-to-ligand charge transfer (MLCT) wavelength of the complexes for the DSSCs application. The high molar extinction coefficients and red shift results obtained from such molecular designs were adduced to the extension of the π-conjugation in the complexes due to the introduction of these various groups.

**Figure 3:**
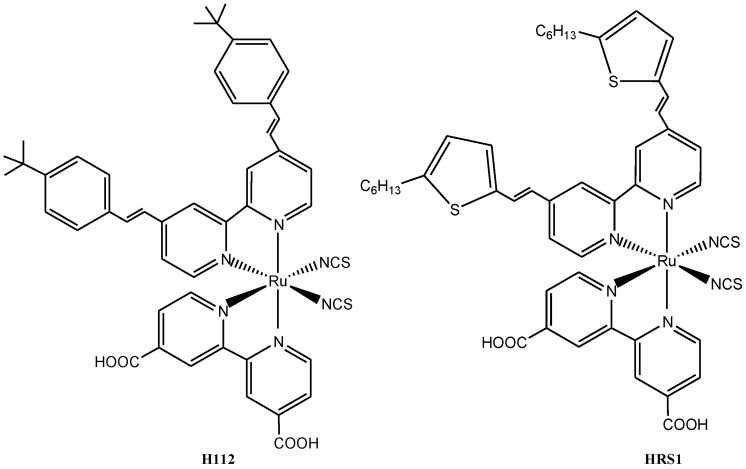
Molecular structures of the ruthenium complexes **H112**, and **HRS1**.

The ruthenium(II) bipyridine complex sensitizer **H112** (Figure 3) showed better sensitivity across the spectral range of 400–800 nm with a molar extinction coefficient value of 16,700 M^−1^cm^−1^ as compared to **N719** (13,200 M^−1^ cm^−1^) under similar conditions, as reported by Chandrasekharan *et al*. [58]. The enhanced molar extinction coefficient of high energy absorption band of **H112** was adduced to the contribution of π→π* transition of 4,4'-*bis*(4-*tert*-butylstyryl)-2,2'-bipyridine to the corresponding metal → ligand charge transfer transition as compared to that of 4,4'-dicarboxy-2,2'-bipyridine in the **N719** complex. When **H112** was compared to **HRS-1**having 4,4'-*bis*((*E*)-2-(5-hexylthiophen-2-yl)vinyl)-2,2'-bipyridine as ligand, a lower molar extinction coefficient of the high energy absorption band was obtained which was found to be due to an increase in the HOMO/LUMO band gap of the molecule. The IPCE spectrum of **HRS1** with relatively higher and broader was found to be similar to **H112** sensitizer in their absorption spectra when adsorbed on titanium dioxide electrode, with values of about 46% and 44%, respectively. However, the IPCE value of **N719** sensitizer was of almost equal to that of **H112**.

Another approach that has been employed to increase the molar extinction coefficient involves the systematic tuning of the LUMO and HOMO energy levels of the ruthenium polypyridyl complexes. On one hand, this approach has been found suitable for the estimation of the optimal threshold wavelength for maximum power conversion of a single-junction converter, and on the other hand, the introduction of a ligand with low-lying π* molecular orbital most especially a strong donor ligand will invariably lead to the destabilization of the metal t_2_g orbital to a lower energy region [59].

**Figure 4:**
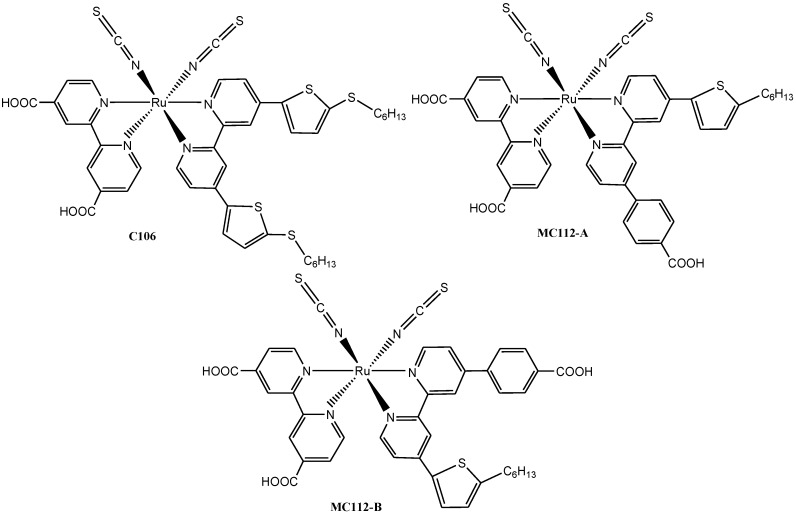
Structures of Ru(II) polypyridyl complexes with thiophene derivatives.

In a recent work reported by Lobello, *et al*., new Ru(II) dyes (**C106, MC112A & B**) (Figure 4) containing (4-(5-hexylthiophen-2-yl)-4'(4-carboxyl-phenyl-2,2'-bipyridine) and 4,4'-dicarboxy-2,2-bipyridine as ligands were prepared and the light-harvesting properties of the dissymmetric mixed bipyridine ligands were examined in order to achieve combination of desired properties and possibly optimum number of carboxylic acid functionality for anchoring on TiO_2_ semiconductor. The UV-Vis and TDDFT-simulated absorption spectra of **MC112** in ethanol solution showed bands at 524, 387 and 307 nm with ε = 18,300, 20,950, and 56,100 M^−1^ cm^−1^, respectively. The main visible transition was found to have an increased molar extinction coefficient compared to **N719** with a slight blue-shift in absorption which was tentatively ascribed to the different dye protonation and the highest occupied orbitals of **MC112** are mainly contributed by the Ru-NCS character [60].

**Scheme 1:**
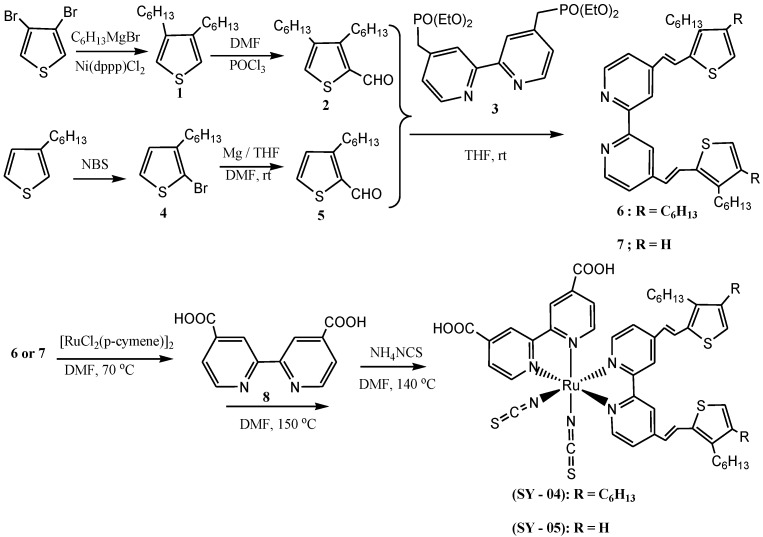
Synthesis of **SY-04** and **SY-05** as reported in literature [3].

Anthonysamy *et al*. reported the synthesis of two heteroleptic ruthenium(II) dyes (compounds **SY-04** and **SY-05**) containing the ancillary ligands of 3,4-dihexylthiophene and 3-hexylthiophene units which were obtained based on the Horner-Emmons-Wadsworth (HEW) reaction method (Scheme 1) [3,60]. The two ruthenium(II) complexes have molar extinction coefficient (ε) of the MLCT band at a peak at 546 nm as 21.7 × 10^3^ M^−1^ cm^−1^, which is higher than that (14.0 × 10^3^ M^−1^ cm^−1^) of **N3**. Base on the argument that for an ancillary ligand containing thiophene, the introduction of alkyl chains was found to increase the solubility. However, some studies have shown that the position of attachment of alkyl chains on the thiophene substituted 2,2-bipyridyl ligands often affect their solubility indifferent polar solvents [61,62].

**Figure 5:**
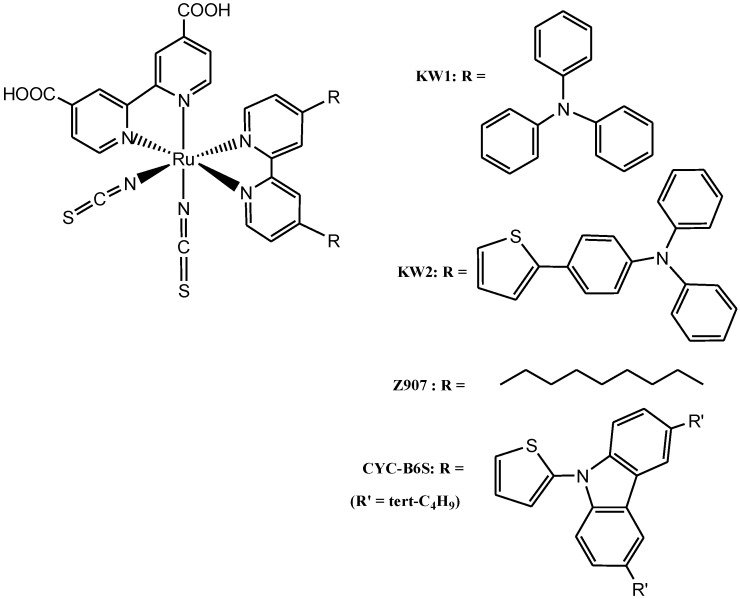
Molecular structures of the ruthenium complexes **KW1**, **KW2**, **Z907** and **CYC-B6S**.

Cao *et al*. [63] recently investigated the influence of two electron-donating antennas on the photophysical and photo-electrochemical properties of some ruthenium complexes. The molecular engineering of these complexes was accomplished using triphenylamine and thiophene substituted triphenylamine in which thiophene was used as spacer between the triphenylamine and the bipyridine units. The two new heteroleptic ruthenium sensitizers (**KW1** and **KW2**) (Figure 5) exhibit lower energy MLCT bands centred at 538 and 554 nm, which were respectively, 18 and 34 nm red-shifted compared to the reference **Z907** dye. The molar extinction of **KW2** (24.3 × 10^3^ M^−1^ cm^−1^), was found to be much better than that of **KW1** (18.4 × 10^3^ M^−1^ cm^−1^), **Z907** model dye (12.0 × 10^3^ M^−1^ cm^−1^) and the **CYC-B6S** dye (16.1 × 10^3^ M^−1^ cm^−1^) [64].

Complexes **KW1** and **KW2** exhibit remarkable light harvesting capacities with impressive power conversion efficiencies close to 11% under AM 1.5G simulated sunlight. Thiophene as conjugated spacer has been demonstrated to have a profound effect on the photophysical properties of [Ru(tpy)_2_]^2+^ complexes because of its polarizability and low resonance energy [65].

**Figure 6:**
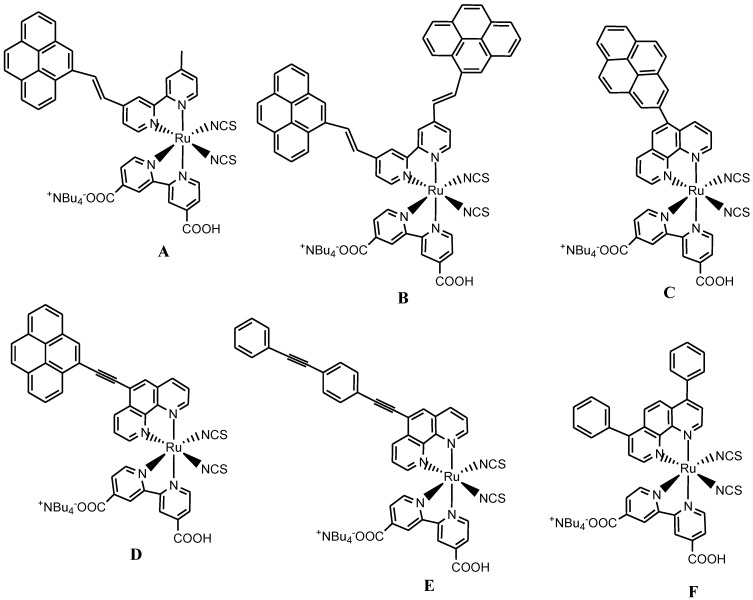
Representative structures of [Ru(bpy)_n_(L)_m_(NCS)_2_]^2+^ analogues [66].

**Scheme 2:**
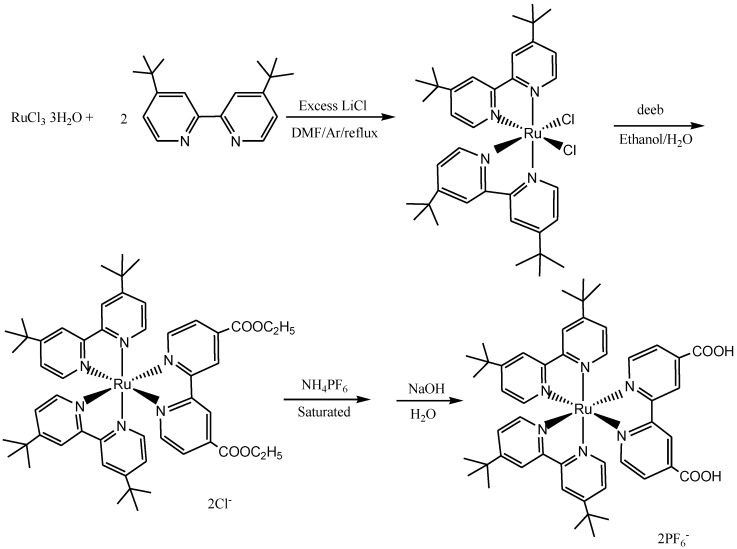
Representative synthetic procedures for homoleptic and/or heteroleptic [Ru(bpy)_3_]^2+^ analogues [66].

**Scheme 3:**
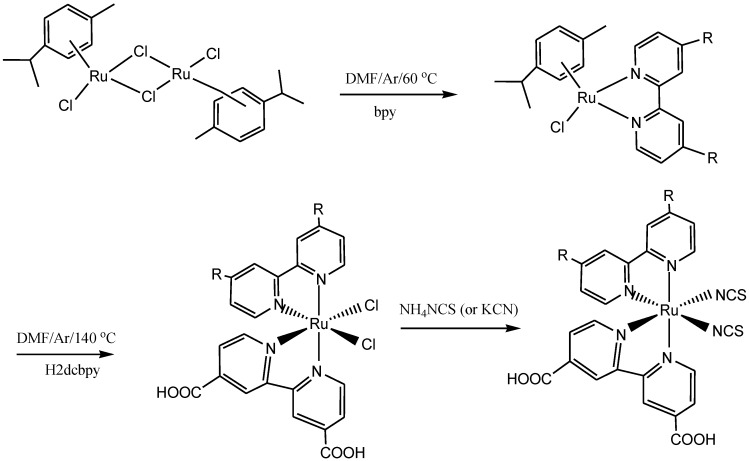
The general synthetic route for homoleptic [Ru(bpy)_3_]^2+^ or heteroleptic [Ru(bpy)_n_(**L**)_m_ (NCS)_2_]^2+^ analogues [66].

In a similar trend, Sun reported several ruthenium(II) synthons or dyes (Figure 6) routinely employed in DSSCs using both the conventional (Scheme 2 and Scheme 3) and microwave irradiation synthetic techniques [66]. Unlike conventional heating, the microwave method of synthesis has the general ability to superheat the solvent in a closed reaction vessel and hence shorten the reaction time, and most often this results in high product yields which normally comes as a result of minimal purification and work-up steps. However, despite of the versatility of the microwave synthetic method, Durham and co-workers [67] in their report observed that microwave synthesis may not be suitable for all compounds and in particular, for compounds containing ester groups due to the operating system of microwave reactors. A change in solvent properties may likely prevent decarboxylation of dyes with esters or carboxylic acid groups if low boiling point solvents are used. This showed that appropriate reaction conditions are invariably needed for certain Ru(II) chromophores intended for solar cells thus confirming some of the limitations of this method.

### 2.1. Tuning of the Excited State Properties of Ru(II) Polypyridyl Complexes

Photoactive materials have been found most useful as coordination complexes to mimic components of artificial photosynthetic devices. The excited state lifetime is a very important property of a photosensitizer. If the photosensitizer is to function properly it must be long enough to provide the desired electron and energy transfer. Many different strategies used at extending the excited state lifetime have proven successful such as the use of electron donating or withdrawing substituent’s [68,69]. Strong electron withdrawing groups such as SO_2_Me attached to the 4'-position of the terpyridine gives room temperature excited state lifetimes of around 30 ns [70]. However electron donating groups will destabilize the metal based HOMO more than they destabilize the ligand-based LUMO and therefore non-radiative decay will be facilitated. Electron withdrawing substituents will instead lower the ^3^MLCT excited state energy due to a greater stabilization of LUMO as compared to the metal-based HOMO [69].

An increase in the excited state lifetime could also be achieved by the use of bichromophoric systems, with an intrinsically long-lived organic triplet at a somewhat lower energy than the ^3^MLCT state, serving as an energy reservoir. If this strategy is to result in a longer excited state lifetime the electronic coupling between the different chromophores must be minimized so that the individual electronic properties are maintained. This approach often results in very long excited state lifetimes but bi-exponential decays are frequently observed. The gain in emission lifetime is however exactly counterbalanced by a loss in reactivity because the fractional population of the MLCT state is correspondingly small. Thus the yield of photoreaction from the MLCT state would not increase by this approach, as demonstrated by the fact that the MLCT emission yield is as low as in the reference complexes without organic chromophore. Furthermore, the addition of the extra chromophore will also destroy the possible construction of linear rod like molecular arrays [71].

**Figure 7:**
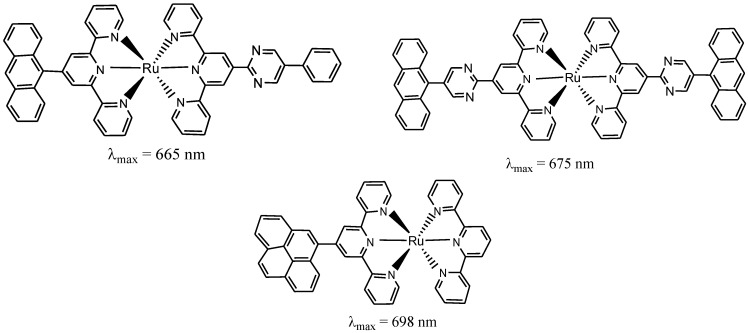
Structures of Ru(II) polypyridyl complexes with no anchoring groups.

In support of this view, Medlycot, and Hanan, in their report, showed that the most efficient means of prolonging room temperature luminescence lifetimes is through a bichromophoric system (Figure 7), in which emission from the ^3^MLCT excited state is delayed by equilibration with an isoenergetic triplet state of another chromophore [72].

**Figure 8:**
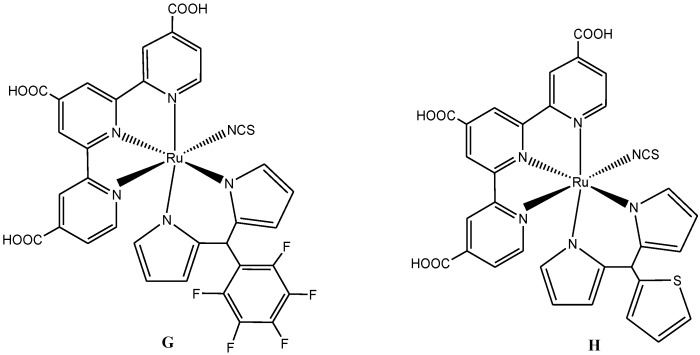
Coordination of bidentate 5-pentafluorophenyldipyrrinate (pfpdp) or 5-(2-thieny) dipyrrinate (2-tdp) to a Ru(II) centre bearing terpyridyl tricarbonate and NCS ligands.

Guocan, *et al*. [73], reported the synthesis and characterization of near-infrared photoresponse properties of some ruthenium dipyrrinate terpyridine sensitizers [Ru(tctpy)(L)(NCS)] (L = pfpdp or2-tdp). The new complexes involve the substitution of bidentate 5-pentafluorophenyldipyrrinate (**G**) or 5-(2-thienyl)dipyrrinate (**H**) for two NCS- ligands in the ruthenium black dye (Figure 8). The deposition of the new complexes [Ru(tctpy)(R-dp)(NCS)] on a mesoporous TiO_2_ electrode has been found to have an incident photon-to-current efficiency up to 950 nm, which is one of the highest values reported so far for molecular sensitizers in dye-sensitized solar cells.

### 2.2. A New Design Strategy to Extend the π-System of Ru(II) Bi- and Tri- and Terdentate Complexes

As observed from previous sections, it is not an impossible task to increase the excited state lifetime of Ru(II)-polypyridyl complexes based on polypyridyl ligands. However, if all the requirements described must be fulfilled and the driving force for further reactions is high enough, a new approach that does not stabilize the ^3^MLCT state but rather destabilizes the ^3^MC state has to be developed [74,75,76]. The ruthenium(II) polypyridyl complexes containing 2,2'-bipyridine, 1,10-phenanthroline, or 2,2';6'2''-terpyridine ligands, usually have strong absorptions in the visible region with molar extinction coefficients around 10^3^ to 10^4^ M^−1^ cm^−1^. The strong absorption is usually derived from MLCT d-π* transitions. This transition is allowed by the spin selection rule (ΔS = 0) between the orbitals with the same spin multiplicity and the LaPorte selection rule (Δl = ±1) between orbitals with different symmetry. The d-d transitions are forbidden especially for octahedral complexes due to their rigid symmetrical geometry violating the LaPorte selection rule. Even for distorted octahedral complexes, the metal-centred d-d transitions can only have molar extinction coefficients of 1–100 M^−1^ cm^−1^.

The strategy in tuning the excited state properties of the Ru(II) complexes is based on the new design of bidentate, tridentate and terdentate polypyridyl ligands by extending the π-system of the ligands, a strategy which relies on the presence of additional chromophore(s) with a non-emissive, long-lived triplet state of comparable energy to the ^3^MLCT state using alkylene, alkynylene, bis-(pyridyl)triazine, aryl and polyaromatic [72,77].

### 2.3. Synthesis of Bipyridine Derivatives and Related Ru(II) Complexes

**Scheme 4:**
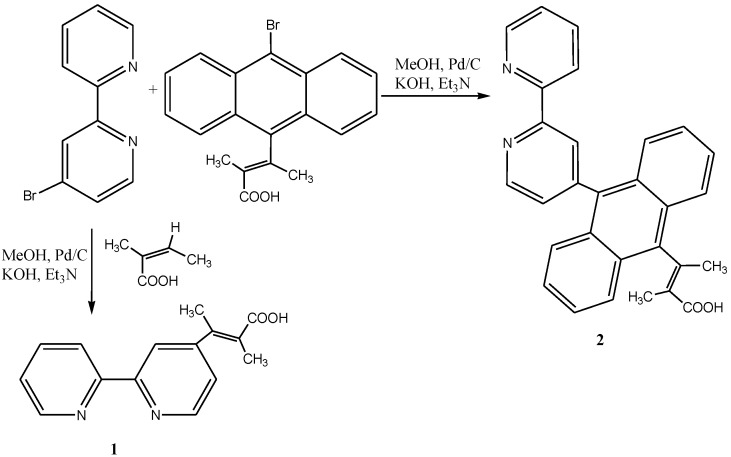
Synthesis of anthracenylbipyridine using palladium/carbide catalyst [75].

Intramolecular charge transfer has been observed in bipyridine ligands bearing electron-donating groups (the bipyridine rings serve as the electron acceptor) such as alkenyl (**1**) and anthracenyl (**2**) moieties. Using palladium-carbide catalysis in a basic medium (Scheme 4), alkenyl and anthracenyl bipyridines were synthesized in good yield.

**Scheme 5:**
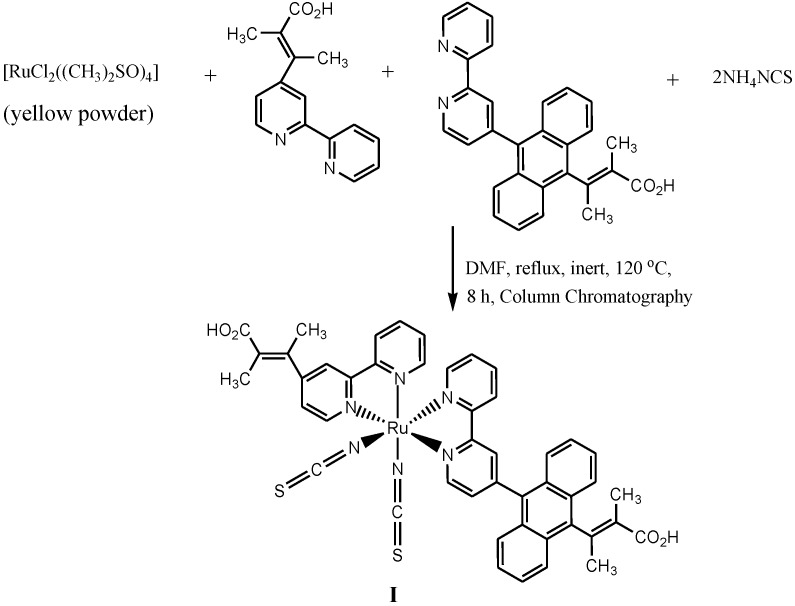
The general synthetic route for heteroleptic [Ru(bpy)_n_(NCS)_2_]^2+^ complex [75].

The synthesis and characterization of Ru(II) complex (**I**) (Scheme 5) obtained when **1** and **2**above were used as coordinating ligands was reported by Adeloye and Ajibade [75]. That work provided important information on the effect of reducing the number of anthracene molecules attached to polypydyl ligands, which has been found to largely affect the absorption properties. A decreased value in the molar extinction coefficients was observed in ruthenium(II) polypyridyl complexes with higher number of attached anthracene moieties [78].

**Figure 9:**
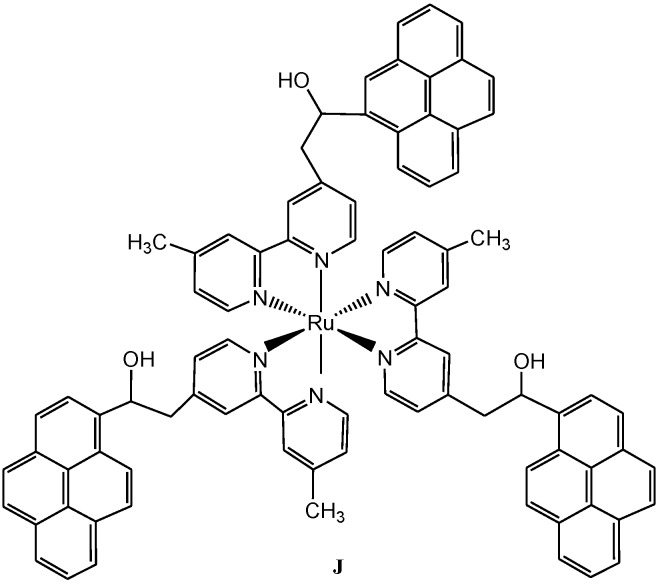
Molecular structure of Ru(II) bipyridine with pyrenyl chromophores (**J**) [79].

Tyson and Castellano [79] reported the synthesis, spectroscopic and photophysical properties of pyrene molecules covalently linked through alkyl tethers to a single ruthenium(II) diimine MLCT complex (**J**) (Figure 9). It was shown that the UV excitation of the pyrenyl antennae results in rapid and efficient singlet-singlet energy transfer to the [Ru(bpy)_3_]^2+^ core, which is the basis for the sensitized MLCT-based emission. It has been established that in cases where two chromophores are separated by an alkyl tether, a reversible triplet-triplet energy transfer occurs between the ^3^pyrene and the ^3^MLCT excited states with the consequence that the observed ^3^MLCT-based emission is longer lived. This observation was ascribed to the imparted stabilization of the equilibrium process. However, the triplet metal-to-ligand charge transition may become extremely long in the absence of spacer group between the diimine and the pyrene unit.

**Figure 10:**
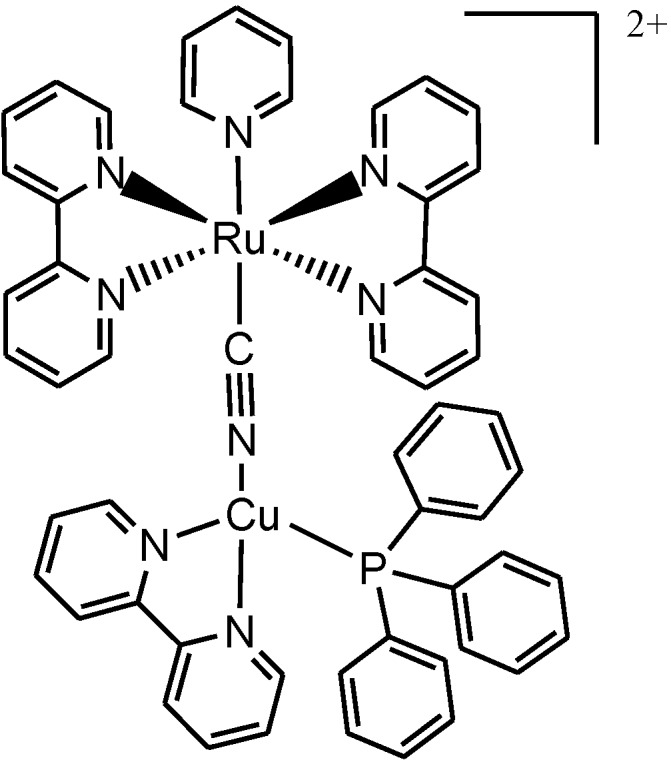
The structure of [(bpy)(PPh_3_)Cu(NC)Ru(bpy)_2_(py)]^2+^.

In 2002, Ranjan and Dikshit [80], reported the preparation, photophysical and electrochemical behaviour of new binuclear complexes with [Cu(PPh_3_)_3_]^+^, [Cu(PPh_3_)(*N*˗*N*-2,2'-bipyridine, 1,10-phenanthroline) moieties connected via the isocyanide group to [Ru(bpy)_2_(py)]^+^ and [Ru(phen)_2_(py)]^+^(PF_6_)_2_. Inaddition, new trinuclear complexes [{(PPh_3_)_3_Cu(µ-NC)}_2_Ru(bpy)_2_](PF_6_)_2_ and [{(*N*˗*N*)-(PPh_3_)Cu(µ-NC)}_2_Ru(bpy)_2_](PF_6_)_2_ were also reported. The complexes showed appreciable luminescence with emission wavelengths in the 458–550 and 600–636 nm ranges. It was shown that the isocyano-bridged complexes are more powerful excited state reductants than the cyano-bridged, Cu^(I)^(µ-CN)Ru^(II)^ complexes (Figure 10) [80].

**Scheme 6:**
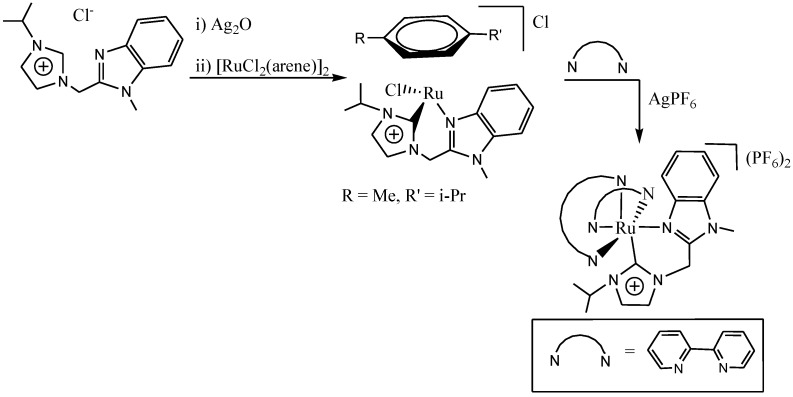
Synthesis of ([Ru(bpy)_3_]^2+^ analogues containing an *N*-heterocyclic carbine ligand.

Wadih and co-workers [81] reported the synthesis of novel ruthenium(II) complexes comprising an *N*-heterocyclic carbene and ancillary polypyridine ligands from the corresponding Ru(NHC)(arene) precursors via arene displacement which was found to be dependent on the starting material and the polypyridine used (Scheme 6). The arene substitution reaction is of considerable scope as a variety of carbene ruthenium precursors are known and readily accessible. The NHC-modified ruthenium polypyridine complexes were found to display beneficial optical and electrochemical properties which may endear them as potential photosensitizers in dye-sensitized solar cells.

### 2.4. Phenanthroline and Its Derivatives: Introduction and Basic Transformations

#### 2.4.1. Halogenation

In the recent times, it has been shown that the π-acceptor and σ-donor strengths of the polydiimine ligand, solvent, and temperature are the major primary importance factors that drive both the redox and excited-state properties of ruthenium(II) polypyridine complexes [82]. Thus, it becomes imperative to systematically design complexes with specific ligand functionality that could absorb at the wavelength of choice and are also capable of channelling the energy onto different ligands.

**Scheme 7:**
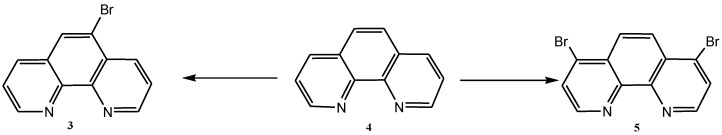
Synthesis of 5-bromo-1,10-phenanthroline and 4,7-dibromo-1,10-phenanthroline.

Halogenated derivatives of 1,10-phenanthroline for example, compounds **3** and **5** (Scheme 7) have served as point of convenience or starting reagents for the synthesis of more elaborate ruthenium(II) polypyridyl complexes. Brominated phenanthrolines have also served as convenient substrates for palladium-catalyzed alkynyl and aryl coupling reactions.

#### 2.4.2. Alkylation and Catalyzed Cross-Coupling

**Scheme 8:**
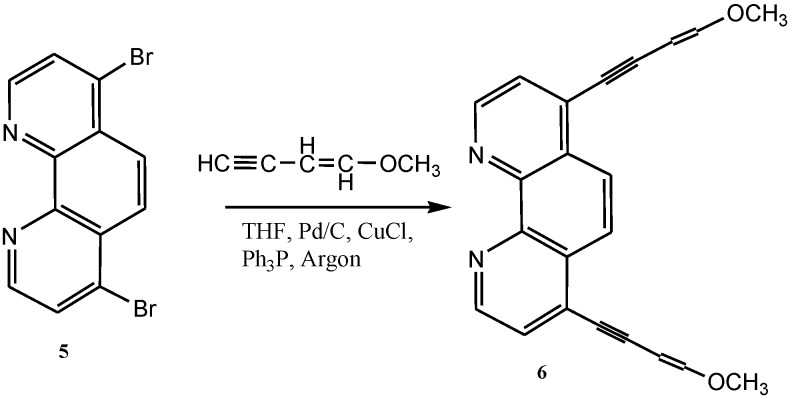
Synthesis of 1,10-phenanthroline containing functionalized alkynyl derivatives.

The addition of alkyl [83] and aryl [84,85] groups to the 2- and 9-positions of 1,10-phenanthroline proceeds most commonly by using organolithium reagents. Alkyl extension of methylated-1,10-phenanthroline is also achieved by lithiation [86]. Ziessel and co-workers were instrumental in the application of palladium(0)-catalyzed coupling reactions to bromo- and chloro-substituted phenanthrolines, bipyridines, and terpyridines [87,88]. In all cases, conditions have been established to promote the coupling of various alkynyl (**6**) moieties to these ligands, making it possible to generate new materials such as rod-like organometallic structures and polymers (Scheme 8) [89,90].

**Figure 11:**
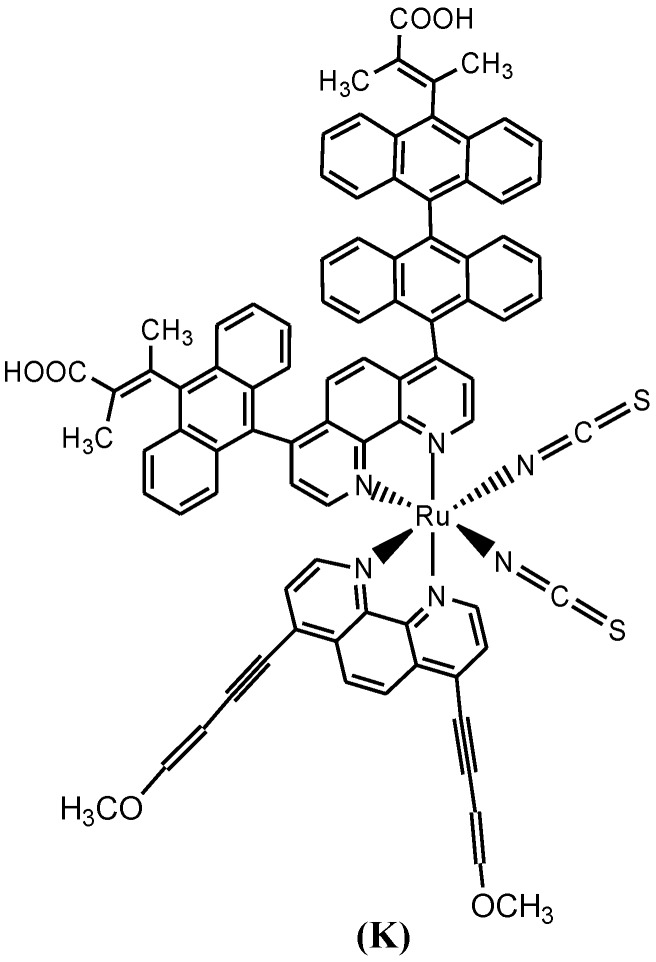
Molecular structure of Ru(II) phenanthroline ligand with alkynyl and anthracenyl substitutions.

In relation to compound **6** above, Adeloye and Ajibade [90] reported two series of bidentate phenanthroline ligands coordinated to ruthenium. The resulting Ru(II) complex (**K**, Figure 11) had characteristic broad and intense metal-to- ligand charge transfer (MLCT) bands absorption in the visible region (λ_max_ = 461 nm, ε = 41,400 M^−1^cm^−1^). It was envisioned that the extension of the π-conjugation using anthracene and 1,3-enyne moieties on phenanthroline ligand may lead to enhance incident photon to current efficiency (IPCE) in the dye-sensitized solar cells.

**Scheme 9:**
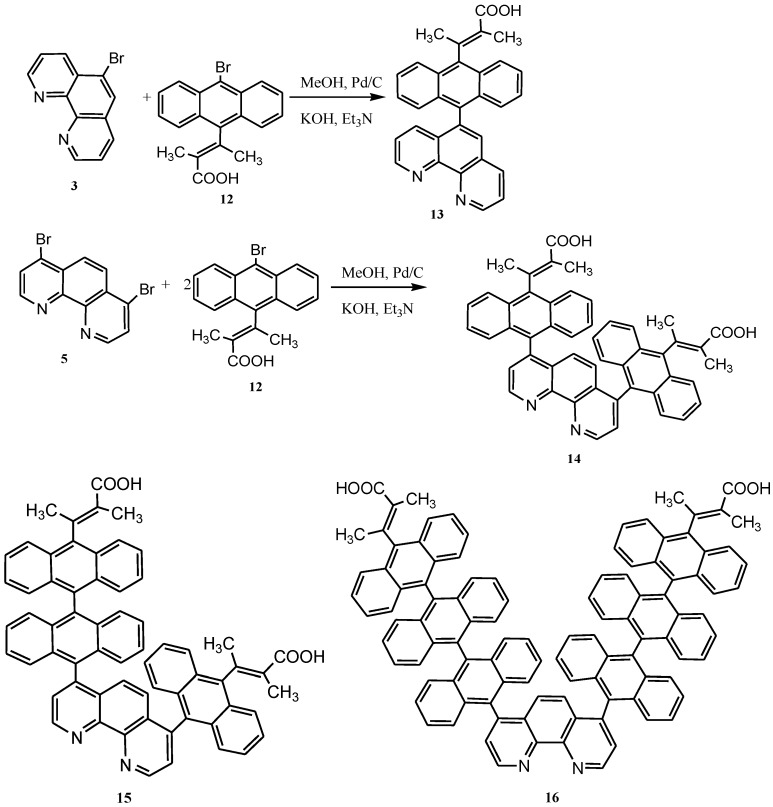
Cross-coupling reactions of two arylhalides using palladium-carbide as catalyst.

A recent study showed the preparation of various derivatives of 5- and 4,7-disubstituted 1,10-phenanthrolines with 9-bromoanthracenyl-10-(2-methyl-2-butenoic acid) (**12**) to form single (**13**), single-single (**14**), single-double (**15**), and triple-triple linked (**16**) anthracenyl derivatives in a palladium-carbide catalysis involving cross-linking of two aryl-halides (Scheme 9). The rich coordination chemistry of 1,10-phenanthroline has encouraged the synthesis of new structures that serve as electron acceptors when chelated to appropriate metal complexes. Schanze and Sauer prepared a variety of proline-bridged *p*-benzoquinone derivatives of [Ru(bpy)_2_(phen)]^2+^, connected to the metal centre through an amide bond utilizing the carboxy terminus of l-proline [91]. In all cases, the amide linkage to l-proline was generated from the phen-NH_2_ group resident on the metal complex.

**Figure 12:**
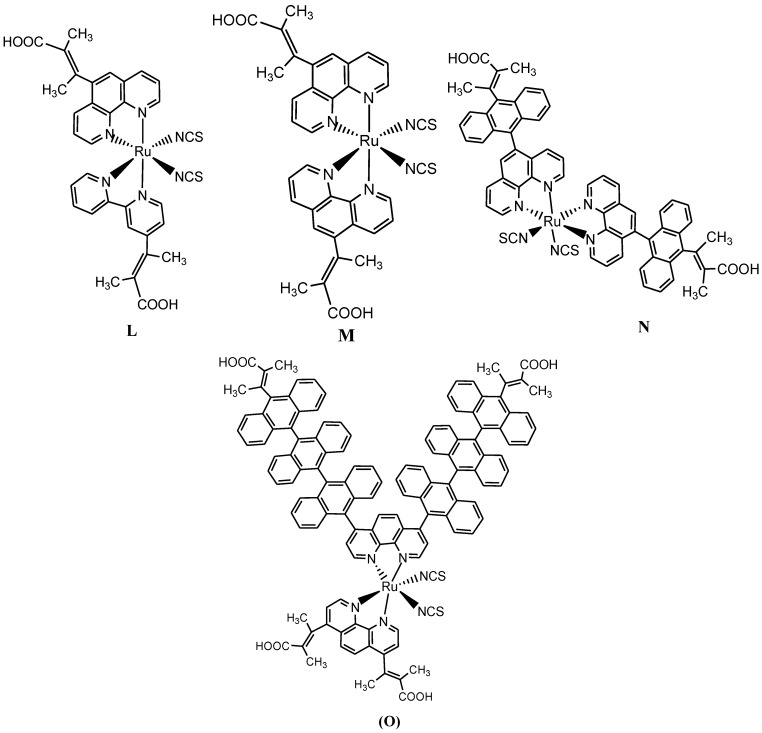
Representative structures of mixed ligand and/or heteroleptic [Ru(phen)_n_(**L**)_m_(NCS)_2_]^2+^ analogues.

Ruthenium(II) complexes having bipyridyl and/or phenanthrolyl ligands functionalized with one to three anthracene units have been reported and studied to determine the effect of increased π-conjugation on the photophysical and electrochemical properties (Figure 12). The synthetic route followed two step pathways which involve initial mono- or dibromination of the starting polypyridyl ligand. A one-step nucleophilic aromatic substitution reaction of the aryl bromide compounds and anchoring ligand was done in a basic reaction medium under palladium catalysis to afford the functionalized ligands. Sequential substitution of the DMSO coordinating ligand from the metal precursor with stoichiometric ratio of the ligand with metal precursor and subsequent work-up with chloride exchange with thiocyanate afford the desired ruthenium(II) functionalized polypyridyl complexes. Anthracene derivatives have been used for the detection of high energy photons, electron, alpha particles and showed both hole and electron transport and hole-transfer materials. Due to π-electron cloud overlaps, anthracene exhibits semiconductor properties. Organic semiconductors have some merits of self-radiation, flexibility, light weight, easy fabrication and low cost. These materials have also been investigated as organic electroluminescence materials for the applications in organic solar cells. The observed trend in the UV-Vis absorption will be for the chromophore (anthracene) to absorb all the wavelength coverage concomitant with good molar extinction coefficient at the near visible region (300–405 nm) that covers the vibronic peaks for the intra-ligand (π→π*) charge transfer transitions characteristics of anthracene derivatives, which may compliment the ^1^MLCT absorption of the metal complex for a better photon absorption and possibly enhance the IPCE values of the dye-sensitized solar cells [87,90,92].

#### 2.4.3. Molecular Recognition and Phenanthroline-based Ionophores

Some of the most successful sensing applications of 1,10-phenanthroline and its derivatives are realized when they are chelated to a ruthenium(II) centre. The visible-absorbing metal-to-ligand charge transfer (MLCT) excited states associated with these complexes possess long-lifetimes and high quantum yield photoluminescence [93]. The long lifetimes associated with these chromophores make them susceptible to collisional quenching reactions, such as electron and energy transfer. In terms of sensing, the orange-to-red MLCT-based emission provides a stable and accurate response (in intensity and lifetime) to dioxygen. Demas and co-workers have thoroughly developed this idea using a variety of ligands, metal centres, and solid support materials [94]. The charge transfer photoluminescence in ruthenium(II) diimine complexes is also temperature dependent, providing a luminescence response that can accurately determine temperature in a variety of environments [95].

#### 2.4.4. Chromophore-containing Phenanthrolines

**Figure 13:**
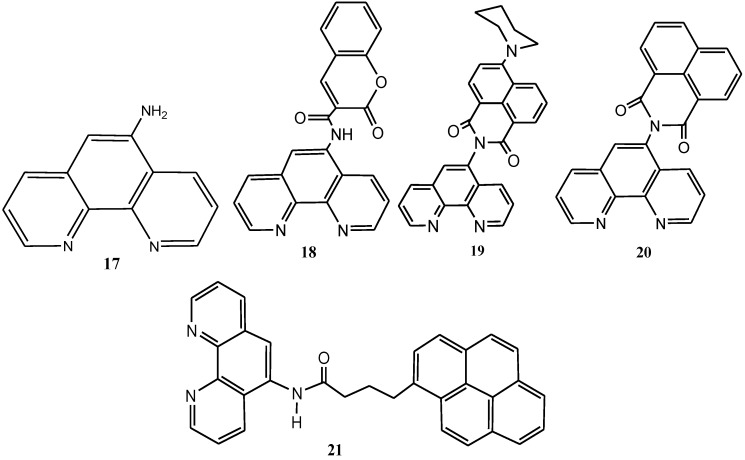
Molecular structures of 5-position functionalized phenanthroline chromophores.

1,10-Phenanthroline and its derivatives have been largely unexplored for chromophore attachment unlike the 2,2'-bipyridine and 2,2',6'2"-terpyridine structures since there are a limited number of reactive structures available for synthetic elaboration. Luman and his group have made use of nucleophile **17** in the preparation of coumarin and naphthalimide-containing ligands **18**, **19**, **20** (Figure 13) [79,96,97]. The introduction of these organic chromophores into ruthenium(II) complexes has led to MLCT compounds possessing large absorption cross-sections and markedlyextended room-temperature, excited-state lifetimes. Rodgers and co-workers have also made use of **17** as a nucleophile in the preparation of an amide-linked pyrene derivative of 1,10-phenanthroline (**21**) [98]. [Ru(bpy)_2_(**21**)]^2+^ served as the basis for future studies of excited triplet-state equilibria in ruthenium(II) MLCT chromophores.

### 2.5. Chemistry of 2,2':6',2"-Terpyridine and Related Ruthenium(II) Complexes for DSSCs

Isolation of terpyridine was first reported by Morgan and Burstall in the early 1930s [99]. The coordination to a metal ion like ruthenium- to this tridentate ligand was reported to impose a small bite angle and consequently a ligand-field strength that is lower than that of bpy [100]. This behaviour allows thermal access to low-lying non-emissive metal-centred (MC) states, and the MLCT excited-state is short lived (0.25 ns for [Ru(tpy)_2_]^2+^ [101]. The ever-expanding potential applications are the result of advances in the design and synthesis of tailored terpyridine derivatives. The well-known characteristics of terpyridine metal complexes are their special redox and photophysical properties, which greatly depend on the electronic influence of the substituents. Therefore, terpyridine complexes may be used in photochemistry for the design of luminescent devices [102], or as sensitizers for light-to-electricity conversion [103]. Ditopic terpyridinyl units may form polymetallic species that can be used to prepare luminescent or electrochemical sensors [104]. Another interesting application regarding novel supramolecular architectures is the formation of “mixed complexes”, where two differently functionalized terpyridine ligands are coordinated to single transition metal ion [105].

#### Cross-Coupling Synthesis of Terpyridine Derivatives

Appropriate methodologies for the construction of functionalized terpyridines are based on directed cross-coupling procedures. Traditional examples, such as the cross-coupling of organosulphur compounds [106,107] or lithiopyridines with CuCl_2_ [108], have the disadvantage that they generally result in overall poor conditions.

Modern Pd(0)- catalyzed coupling reactions combine the desired efficiency and simplicity with controllable substitution possibilities. Suzuki [109], Negishi [110], and Stille couplings [111] are all based on a Pd(0)/Pd(II) catalytic cycle. Substitution at the 4'-position of tpy is unique in that it provides a ligand which still retains the *C*_2_ symmetry of the parent molecule.

**Scheme 10:**
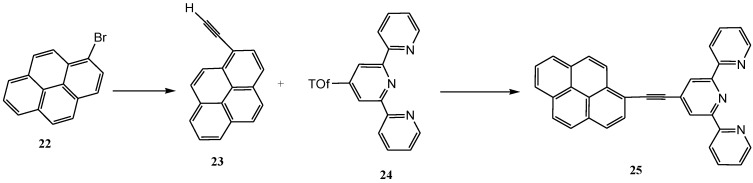
Palladium(0) promoted cross-coupling synthesis of ethynyl-pyrenyl terpyridine ligand [111].

**Scheme 11:**
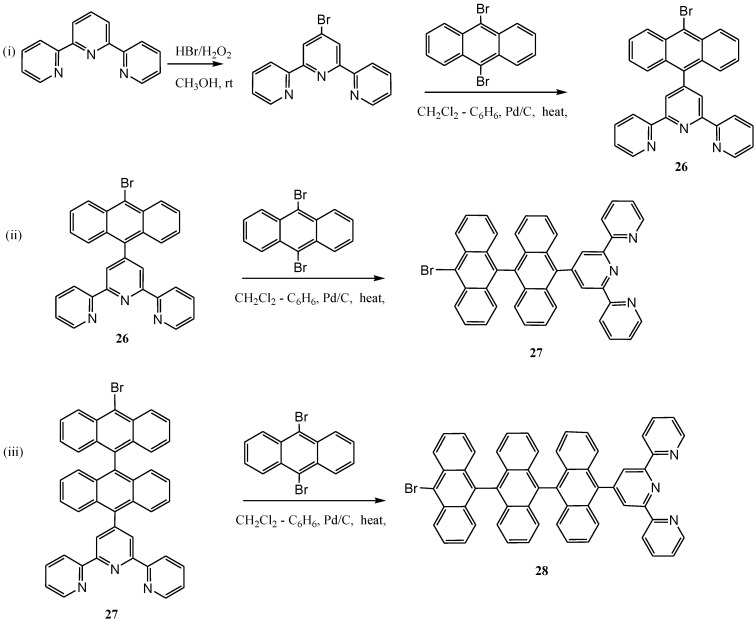
The synthesis of mono-, di and tri-anthracenyl functionalized terpyridines [112].

As shown in Scheme 10 and Scheme 11 respectively, terpyridine ligands were functionalized at 4'-position with ethynylene-pyrene and an increasing number of anthracene. Studies on their corresponding ruthenium(II) complexes have also been reported [112,113].

Since bichromophoric molecular systems in recent developments give insight into the mechanisms of light-induced energy and are photosensitizers that are able to absorb in the near IR-region most especially in the present day dye-sensitized solar cells, it has been shown that extension of the region of ICPE spectra sensitivity with black dye or its derivatives from the present 500–600 nm towards 700–900 nm would lead to increase in the J_SC_ from 20 to 28 mAcm^−2^ for overall efficiency of 15%. Towards achieving this goal, long-range electron transfer in artificial systems and as photosensitizers has been found as a useful means of extending the photon absorption of molecular dyes. The ability of a molecule to switch between off and on states is one of its most important applications. This includes charge transfer over distances greater than 10 Ǻ in both covalent and non-covalent donor-bridge-acceptor systems. For example, in a system with a moderate relaxation rate, the pulse chirping has been shown to increase the efficiency of electron transfer from the donor to the acceptor state [114,115,116,117]. Inaddition, it has been reported earlier that singlet excitation energy is often transferred to appended ruthenium(II) complexes when covalently bonded to naphthalene, pyrene or anthracene units. The nature of polycyclic chromophore dictates the identity of the lowest energy triplet state [118].

**Scheme 12:**
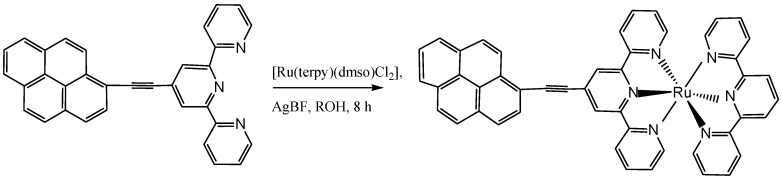
Synthetic profile for Ru(II) bis(terpyridyl) complex containing alkynylpyrene moiety [112].

Hissler, *et al*. [112] investigated spectroscopically the energy-transfer processes in geometrically constrained dyads having ethynylene, pyrene, and ruthenium(II) tris(2,2'-bpy) terminals in which the terminals are intended to retain relative energy levels with connectors having different propensity for through-bond or through-space energy transfer. The results were compared to the corresponding ruthenium(II) /Os(II) bis(2,2':6',2"-terpyridine) complex (Scheme 12) in order to confirm the derived triplet energy spacings. The conclusion drawn from the work showed that though, pyr-Ru acts as a molecular dyad with discreet terminals, there was no indication that solvation dynamics or vibronic processes control the rate of triplet energy transfer in terms of a nonadiabatic process [119,120].

**Scheme 13:**
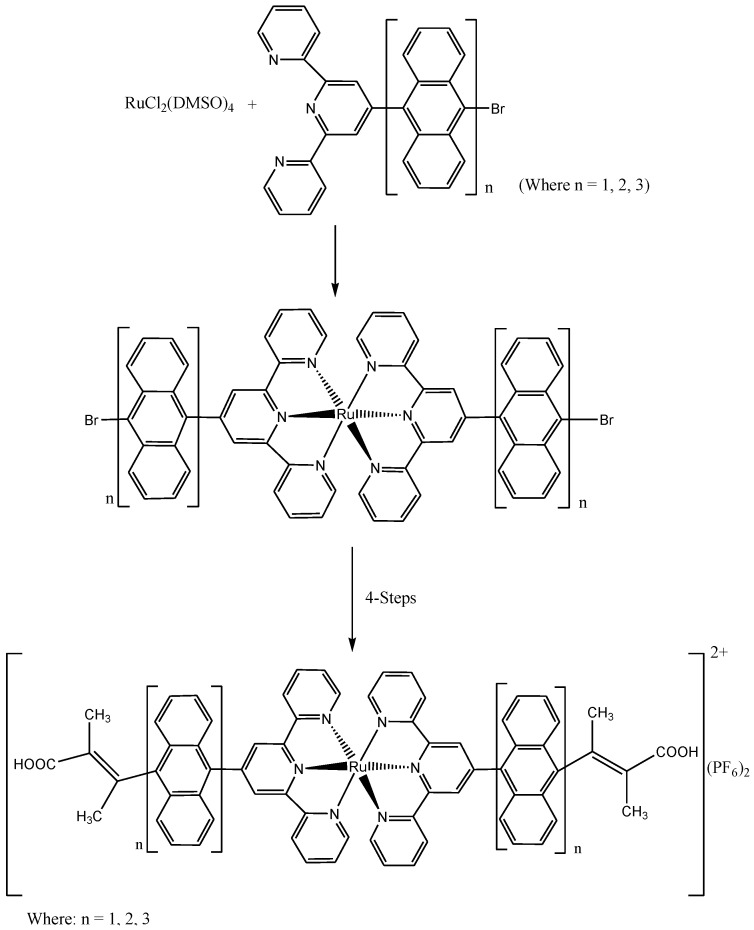
The general synthetic route for preparation of novel homoleptic Ru(II) bis(terpyridyl-oligo-anthracene) complexes [121].

Recently, Adeloye and Ajibade [121] reported the synthesis and characterization of a series of new homoleptic π-conjugated ruthenium(II) bisterpyridine complexes bearing one to three anthracenyl units sandwiched between terpyridine and 2-methyl-2-butenoic acid group (Scheme 13). The general linear correlation between increase in the length of π-conjugation bond and the molar extinction coefficients was investigated. The Ru(II) terpyridine complexes show characteristic broad and intense metal-to-ligand charge transfer (MLCT) band absorption transition between 480 and 600 nm, ε = 9.45 × 10^3^ M^−1^cm^−1^, and appreciable photoluminescence spanning the visible region. In all the complexes, weak shoulder bands were observed in the long wavelength tail region of the absorption spectra at 916 nm (ε = 1.00–1.80 × 10^3^ M^−1^cm^−1^) and 1012 nm (ε = 0.90–2.20 × 10^3^ M^−1^cm^−1^) which were assigned to the triplet metal-to-ligand charge transfer (^3^MLCT) transition.

**Figure 14:**
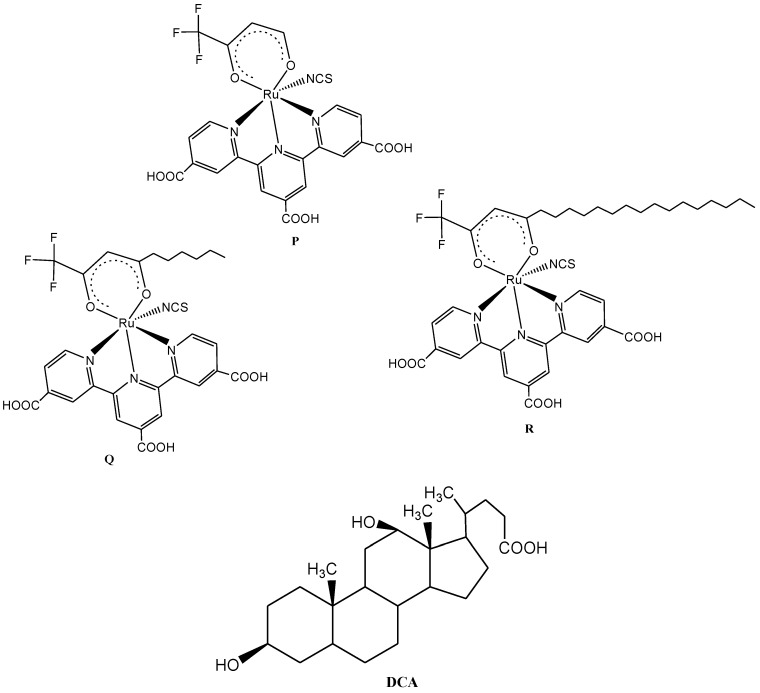
Molecular structures of Ru(II) terpyridyl complexes with/without bulky alkyl chains and deoxycholic acid.

However, in an attempt to investigate the effect of bulky alkyl chain substituents on photovoltaic performances, Islam *et al.* [122] reported the synthesis of three alkyl-substituted β-diketonato-ruthenium(II) polypyridyl sensitizers having different alkyl chain lengths (Figure 14). Although, the reported complexes showed appreciable wavelength absorption spanning the whole visible range extending into the near-IR region (≈950 nm) with gradual enhanced photovoltaic performance as the alkyl chain length increases. However, only the complex **R** with an alkyl chain length of C_16_ was found to show better photovoltaic performance compared to those bearing C_6_ chain lengths (**Q**) and one without an alkyl chain (**P**) irrespective of the presence of deoxycholic acid when anchored to nanocrystalline TiO_2_ semiconductor. The other two complexes **P** and **Q** only gave 10% and 24% enhancement in the presence of deoxycholic acid. The structural nature of the bulky alkyl functionalized terpyridyl complex **R** was adduced to its excellent photovoltaic performance. However, in order to obtain Ru(II) complexes with more interesting photophysical properties, electron withdrawing or donor substitutents on terpyridine would lead to increase in energy gap between the ^3^MLCT and the ^3^MC states [123].

Compared to the vast amount of information and investigations on bpy-based DSC dyes, other ligands containing three or four conjugated pyridine rings in the 2,2'-positions have been scantly reported. This was mainly due to the more difficult synthetic access, considering that the insertion of more than two azine rings in the polypyridine ligand provides only beneficial effects. Substituted quaterpyridines can play a crucial role in the fabrication of efficient dye-sensitized solar cells thanks to the unique panchromatic properties, ranging from UV-Vis to NIR, of their metal complexes. Unfortunately, the difficult synthetic access (low yields, restricted quantities, low reproducibility) and the use of toxic organotin reagents of the so- far reported Stille cross-coupling synthetic access has greatly limited this important potential [124].

### 2.6. Chemistry and Synthesis of Quaterpyridyl Ligands and Related Ruthenium(II) Complexes

**Figure 15:**
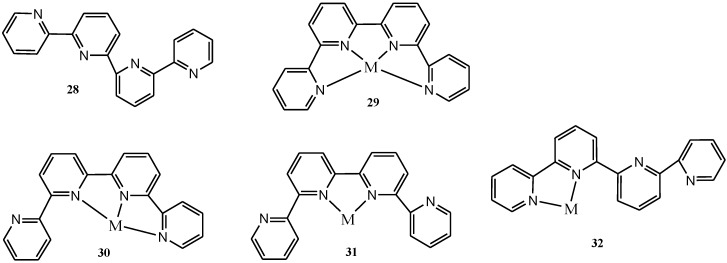
The different structural configurations of quaterpyridyl ligand and modes of coordination to metal ion.

The ligand quaterpyridine (qtpy) is planar with a *trans*, *trans*, *trans* conformation in the solid state **28** [125] and could act as a planar quaterdentate **29**, a terdentate with a non-coordinated pyridine **30**, or as a bidentate with either two non-coordinated pyridines **31** or a non-coordinated bipyridine **32** (Figure 15). The planar quaterdentate mode is found to favour metal ion with preference for octahedral or square-planar geometry. In the case of an octahedral centre, the ligand will occupy the four equatorial sites. This is indeed the case, and majority of complexes of qtpy contain a near-planar ligand in such an environment [126,127,128].

A large number of mono- and dihalogenated pyridines are commercially available for the preparation of BPYs by coupling routes are commercially available. At times, dihalides may not be synthesized through direct lithiation of a suitably activated pyridine with LDA followed by halogenation of this lithiated materia1. Cross-couplings of dihalopyridines may be made to be selective by the choice of the halogen where the reactivity order is I > Br > Cl and by the realization that α- and γ- positions are more reactive than a β-position when the halides are the same. Invariably, considerable control in the preparation of the crucial central bipyridines is possible.

**Figure 16:**
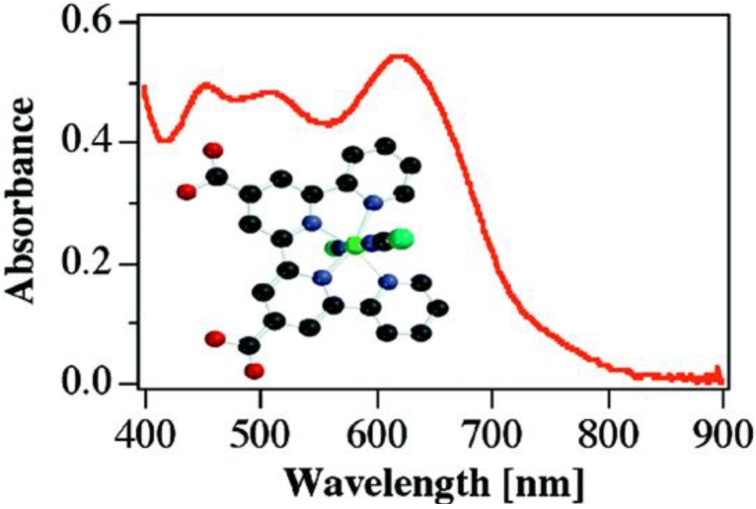
Visible and Near-IR spectrum of ruthenium(II) tetradentate polypyridine ligand [129].

Renouard *et al*. [129] reported the synthesis and characterization of some functionalized tetradentate polypyridine ligands which include: 4,4"'-bis(*tert*-butyl)-4',4"-bis[*p*-(methoxycarbonyl)- phenyl]-2,2':6',2":6",2"'-quaterpyridine, 4',4"-bis[3,4-(dimethoxy)phenyl-2,2':6',2":6",2"'-quaterpyridine and 4',4"-diethoxycarbonyl-2,2':6',2":6",2"'-quaterpyridine. The ruthenium(II) complexes of the *trans*-dichloro and dithiocyanate types obtained using the ligands showed metal-to-ligand charge transfer (MLCT) transitions spanning the entire visible and near-IR regions which endeared them as useful dyes in the dye-sensitized solar cells applications (Figure 16).

Furthermore, it was observed that a blue-shift in wavelengths characterized the absorption and emission maxima of the *trans*-dithiocyanate complexes of the ligands as compared to the *trans*-dichloro analogues was adduced to the strong π-acceptor property of the NCS^−^ compared to the Cl^−^. The incident photon-to-current efficiencies (IPCE) of the complex having 4',4"-diethoxycarbonyl-2,2':6',2":6",2"'-quaterpyridine and (NCS)_2_ as coordinating ligands is much better than that containing Cl^−^at 75% (IPCE) and 18 mA/cm^2^ current density under standard AM 1.5 sunlight.

**Scheme 14:**
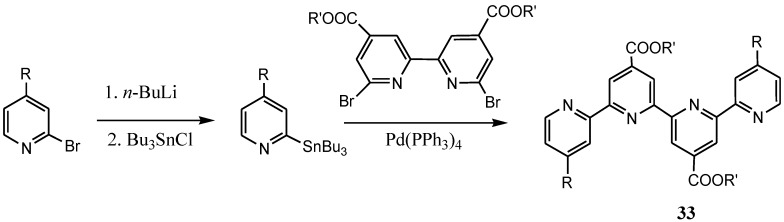
Synthesis of carboxylated quaterpyridine ligands by palladium-catalyzed cross-coupling.

Coluccini *et al*. have also in recent times reported a general synthetic access to carboxylated quaterpyridine ligands **33** of interest for DSC Ru(II) sensitizers, via the Suzuki-Miyaura cross-coupling reaction (Scheme 14). The reaction takes advantage of the higher accessibility and stability, ease of handling and preparation, and low toxicity of boronic acid derivatives, rendering this approach particularly useful for a systematic screening and full-scale production [130].

The synthesis and applications of ruthenium(II) quaterpyridium complexes and poly-*N*-isopropylacrylamide/acrylic acid polymers was reported by Siyambalagoda Gamage [131]. 

**Scheme 15:**
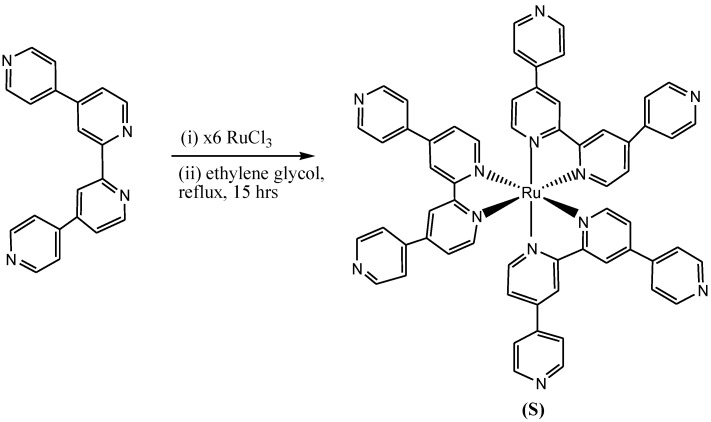
Synthesis of tris(4,4':2',2'':4'',4'''-quarterpyridine-*N''*,*N''*) ruthenium(II) dichloride [132].

Ruthenium(III) chloride and 4,4':2',2'':4'',4'''-quaterpyridine (inexcess) were refluxed for 15 h. The solvent was evaporated by vacuum distillation. After the purification using solvent extraction and recrystallization, pure tris(4,4':2',2'':4'',4'''-quarterpyridine-*N''*,*N''*)ruthenium(II) dichloride (**S**) was obtained. Formation of bis-ruthenium(II) complex was avoided by using excess (4,4':2',2'':4'',4'''-quaterpyridine for the synthesis (Scheme 15).

Moreover, in this report, high pressure and moderate temperature conditions were used to increase the yield of product **S**, but this was found not to affect the reaction yield, and thus it was concluded that heat is a more important factor for the synthesis of tris-homoleptic ruthenium(II) complexes than pressure, and the explanation given to this finding was the existence of alternative binding sites in the 4,4':2',2'':4'',4'''-quaterpyridine ligands, which led to oligoruthenium(II) complexes under high pressure reaction conditions [132].

## 3. Absorption, Emission and Electrochemical Features of Ru(II) Polypyridine Complexes

The molecular structure of the dye affects not only its absorption, photophysical and redox properties, but also the electron-transfer processes occurring at the TiO_2_/dye/electrolyte surface. These parameters strongly determine the overall efficiency of the device. Hagfeldt and co-workers, Juris, and Hans, *et al*. among various authors have reported and documented in great details the photophysical and electrochemical features of typical ruthenium(II) polypyridyl complexes for dye-sensitized solar cells [13,41,133]. At room temperature, the electronic absorption spectra of typical ruthenium(II) polypyridine complexes are often dominated by ligand-based spin-allowed π→π* and n→π* transitions in the UV region of the spectra and by ^1^MLCT absorption bands in the visible region [43,76,83,134]. However, the molar extinction coefficient and λ_max_ value of ruthenium(II) polypyridine complexes at the visible region for the ^1^MLCT absorption band has been found to depend on the type, molecular structure and functionality of the ligand(s) since these involve interplay of the energy gaps and/or transition states. The excited-state properties of ruthenium(II) metal-ligand complexes (MLC) are dependent on the pattern of empty low-lying electronic levels that are ligand dependent, and in particular dependent on the ligand π* energies and the dπ levels of the metal [135].

For mixed ligand Ru complexes, it is therefore possible to some extent to fine tune the photophysical properties by choice of ligands. For example, the use of a ligand with electron-withdrawing substitution such as –COOH or –COOC_2_H_5_ will lead to emission at higher wavelengths because of their lower π* levels, and hence smaller energy gap between the dπ orbitals of the metal center and the π* orbital of the ligand. The use of the ligands that have electron-donating substitutions such as methyl groups will stabilize the hole at the metal center and increase the energy gap, which will increase the quantum yield and lifetimes. The HOMO and LUMO energies provide an estimation of the ionization potential and electron affinity, respectively [59]. Thus, a group with high HOMO energy could be a strong electron donating group, whereas that with low LUMO energy could be a strong electron withdrawing group.

**Figure 17:**
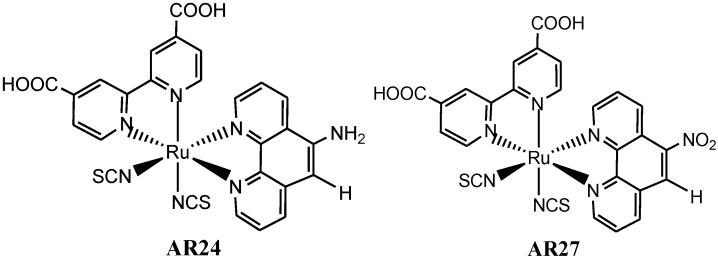
Ruthenium polypyridyl complexes with different ligand substituent’s.

Over the last years, different groups have observed that the presence of certain atoms such as sulphur and oxygen [136] or chemical moieties [137], such as amino (–NH_2_) or nitro (–NO_2_) groups (complexes **AR24** and **AR27** respectively) can have a dramatic influence over the solar cells device output efficiency (Figure 17). Indeed, the presence of certain chemical groups or atoms leads to faster electron-recombination dynamics, which affects the V_oc_ of the device. This observation is similar to the measured effects of the presence of highly conjugated molecular structures in organic sensitizers [138,139] and, thus indicates that the dye molecular structure directly involved in preventing or accelerating the interfacial charge-transfer reaction between the photo-injected electrons and the oxidized species present in the iodine/iodide electrolyte.

Ru(II) polypyridine complexes have shown different electrochemical properties between cathodic and anodic peak potentials. Previous reports on the features of cyclic voltammetry of many complexes of the Ru(II)-polypyridine family is the possibility of localizing with a high degree of certainty the donor or the acceptor orbital of the one electron transfer reaction. Oxidation of Ru(II) polypyridine complexes usually involves a metal centered (parent πM(t_2_g) in octahedral symmetry) orbital, with formation of genuine Ru(III) complexes (low spin 4d^5^ configuration) which are inert to ligand substitution.

[Ru^2+^(LL_3_)]^2+^ ↔ [Ru^3+^(LL)_3_]^3+^ + e^−^

It has been found out that only a one-electron oxidation is obtained in the available potential range. It is not always feasible to compare potentials reported in the literature on an absolute scale. It can be stated that the Ru(II)/Ru(III) potentials in the complexes where the ligands are exclusively true polypyridine molecules, fall in a rather narrow range around +1.25 V with respect to NHE. Reduction of Ru(II) polypyridine complexes may involve either a metal-centered or a ligand-centered orbital, depending on the relative energy ordering. There are several reduction steps in the cyclic voltammetry of the Ru(bpy)_3_ due to strong ligand effect or when the ligand is easily reduced.

### Ru(II) Cyclometalated Complexes for Dye-Sensitized Solar Cells

**Figure 18:**
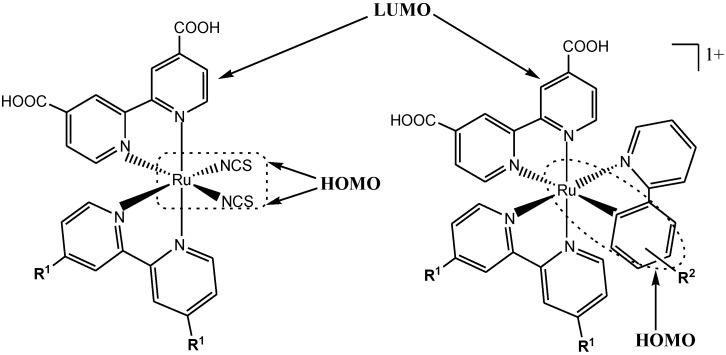
Ruthenium cyclometalated complex obtained through substitution of thiocyanate ligand with phenylpyridine ligand [36].

This review has exclusively focused on the design and synthesis of functionalized polypyridyl ligands; a new insight into this trend is the combination of these polypyridyl ligands with cyclometalating ligands which have been found to influence the photophysical, electrochemical as well as the IPCE of ruthenium dyes used in the dye-sensitized solar cells. Bomben, *et al*. [140,141,142,143], Constable, *et al*. [144], Djukie, *et al*. [145], Albrecht, [146], Bessho, *et al*. [147], Wadman, *et al*. [148] in recent times have synthesized and reported quite a number of ruthenium(II) cyclometalated polypyridyl complexes for light-harvesting dye-sensitized solar cells. A number of chemical properties of the motifs were found to influence the photophysical and electrochemical properties of the resulting complexes. For example, it was found out that the number of unique transitions increases as the anionic character of the ligand shift metal-to-ligand charge transition (MLCT) absorption band to the red region due to cyclometalation which also in effect reduces the symmetry of the point group from *D*_3_ to *C*_1_. In addition, attachment of electron withdrawing groups on the phenyl pyridine ring shifts absorption to the blue-region, whereas an opposite effect was found for electron donating groups. The optical cross section of such dyes increases with addition of conjugated substituents (Figure 18) on the phenylpyridine ring which was adduced to the expansion of the HOMO energy character beyond the metal and phenyl ring, while greater stability could be obtained with substitutions containing aliphatic chains. The ruthenium cyclometalated motifs in general, have been found to satisfy criteria as good chromophores for the dye-sensitized solar cells [36].

## 4. Conclusions

The quest and demand for clean and economical energy sources have increased interest in the development of solar applications where DSSCs have proved to be an alternative approach to the conventional silicon based solar cells. Thus, various methods for the design and development of novel polypyridine ligands with functionalities have been shown and reported, particularly for Ru(II) complexes which can be used as dyes in the dye-sensitized solar cells (DSSCs). Different modes of ligand substitutions were used in the coordination with ruthenium metal center to afford both the homoleptic and heteroleptic types of complexes. Most of the complexes reviewed showed interesting photophysical, photoluminescence and electrochemical properties characteristics of ruthenium(II) complexes that are useful for dye-sensitized solar cells applications.

However, from the UV-Vis absorption data, the molar extinction coefficient values are enhanced majorly at the UV and near-visible region of most compounds as depicted in the stepwise increase in the number of attached π-conjugative bond, but an optimum number of polyaromatic unit with extended conjugation maybe required for a better electronic transfer processes since molecular aggregation do set in which affects the coefficient values and consequently the V_oc_ of the cells. With this important information, it could be established in some complexes that extension of the π-bond conjugation may not be regarded as the only contributory factor for the enhancement of the absorption wavelength to the red region and as well as the molar absorptivity coefficient, but rather, the types and position of substituents on ligands; molecular weight of compounds could as well be adduced to the lowering of these parameters as shown in the overall characteristics displayed by the some of these complexes.

Despite the large absorption and emission wavelengths maxima of many of the reported ruthenium(II) polypyridyl complexes, it could be observed that many of them may not be useful as photosensitizers particularly in application such as the dye-sensitized solar cells due to lack of anchoring ligand groups such as the carboxylate/phosphonate which serves to function as point of attachment on the nanocrystalline TiO_2_ semiconductor.

In addition, most Ru(II) polypyridine complexes containing anthracene probably display better electrochemical properties than those with a perylene moiety. The possible implications of these features may be explained in terms of the different electron transfer processes or mechanisms that are involved between the ruthenium ion, bipyridine, phenanthroline, terpyridine, quaterpyridine, anthracene and/or perylene groups. However, information on density functional theory (DFT) calculations on most Ru(II) polypyridyl complexes used in DSSCs in order to understand the proper relationship between the solar cell performance and the structural properties of the sensitizers is still limited.

This review though, mostly focused on the synthesis and functionalization of polypyridine ligands for Ru(II) complexes, it is in our opinion that apart from the further development and synthesis of novel polypyridine ligands for ruthenium(II) complexation, thorough investigation and understanding of the morphology and compatibility of the ruthenium(II) polypyridine complexes with various semiconductors (*i.e*., TiO_2_, ZnO, SnO_2_, Nb_2_O_5_), electrolytes, and other intrinsic physical features of the working principles of the dye-sensitized solar cells (DSSCs) should be pursued with vigour in the future research in order to enhance the current solar cell efficiency.
